# Prevalence and genotype distribution of human papillomavirus infections among women undergoing HPV testing during 2019–2024 in Xi’an, China

**DOI:** 10.1186/s12879-026-14232-3

**Published:** 2026-08-14

**Authors:** Nan Xu, Li Jin, Burong Li, Yue Zhang, Hua Zhang, Jia Song, Yuqi Zhao, Zhe An, Guodong Li, Xiaoqin Hu, Yanping Zhang, Lun Huang, Weiwei Zhang, Yan Geng, Jianhong Zhu, Fang Huang, Qian He, Bo Cao, Li Xue

**Affiliations:** 1 Department of Clinical Laboratory, The Second Affiliated Hospital of Xi’an Jiaotong University, Xi’an, 710004 China; 2 National-Local Joint Engineering Research Center of Biodiagnosis & Biotherapy, The Second Affiliated Hospital of Xi’an Jiaotong University, Xi’an, 710004 China; 3 Core Research Laboratory, The Second Affiliated Hospital of Xi’an Jiaotong University, Xi’an, 710004 China; 4 Department of Infectious Diseases, The Second Affiliated Hospital of Xi’an Jiaotong University, Xi’an, 710004 China; 5 Key Laboratory of Surgical Critical Care and Life Support (Xi’an Jiaotong University), Ministry of Education, Xi’an, 710004 China; 6 The Second Affiliated Hospital of Xi’an Jiaotong University, 157 West Fifth Road, Xi’an, 710004 China

**Keywords:** Human papillomavirus, Genotype, Prevalence, Cervical cancer, Xi’an

## Abstract

**Background:**

Human papillomavirus (HPV) persistent infection is the primary cause of cervical cancer, with significant regional variations in infection prevalence and genotype distribution. This study aimed to clarify the epidemiological characteristics of HPV infection among women in Xi’an from April 2019 to December 2024, and to provide targeted evidence for local cervical cancer screening and prevention strategies.

**Methods:**

A retrospective analysis was conducted on 33,316 women (aged ≥ 20 years) who underwent HPV testing at the Second Affiliated Hospital of Xi’an Jiaotong University during the study period, with repeated tests from the same individual excluded. PCR-reverse dot blot hybridization was used to detect 28 HPV genotypes. The chi-square test was applied for intergroup comparison of infection rates, with *P* < 0.05 considered statistically significant. Statistical analysis was performed using SPSS 23.0, and graphs were generated with GraphPad Prism 9.0.

**Results:**

The overall HPV positivity rate was 13.96% (4,650/33,316), with single-type infections accounting for 74.67% (3,472/4,650) and multiple-type infections for 25.33% (1,178/4,650). The top three prevalent genotypes were HPV52 (18.00%), HPV53 (12.19%), and HPV16 (9.23%). Significant differences in HPV infection rates were observed among health examination population (12.67%), inpatients (20.85%) and outpatients (37.68%) (*P* < 0.001), as well as across different age groups (*P* < 0.001), with the highest infection rate in the 61–70 years group. The overall HPV infection rate showed a fluctuating upward trend from 2019 (11.52%) to 2024 (19.94%), with high-risk HPV (HR-HPV) infection rate increasing from 7.72% to 15.58% over the same period.

**Conclusions:**

Single-type HPV infection is the main type of infection among women in Xi’an, with HPV52, HPV53 and HPV16 being the dominant genotypes. Infection rates vary significantly by patient source and age, with older women aged 61–70 years at the highest risk of infection. These findings highlight the need to optimize local cervical cancer screening strategies, strengthen HPV vaccination for young women, and conduct regular regional HPV epidemiological surveillance.

## Introduction

Cervical cancer is one of the most common malignant tumors threatening women’s health worldwide, ranking fourth in incidence and mortality among female cancers, with a growing incidence and a younger onset trend [1]. In China, cervical cancer has the highest incidence among female reproductive system malignant tumors, with approximately 110,000 new cases and 59,000 deaths reported annually [2, 3]. Persistent infection with high-risk human papillomavirus (HR-HPV) is the confirmed primary risk factor for cervical cancer and its precancerous lesions, with more than 90% of cervical cancer patients infected with HR-HPV, among which HPV16 and 18 account for about 70% of cases globally [4, 5].

HPV is an epitheliotropic double-stranded DNA virus, with more than 40 genotypes associated with genital tract infections [6]. Per the classification criteria released by the International Agency for Research on Cancer (IARC), HPV genotypes are stratified into high-risk and low-risk human papillomavirus types based on their distinct oncogenic potentials (LR-HPV for low-risk human papillomavirus) [7]. LR-HPV mainly causes benign genital lesions, while HR-HPV is closely related to the occurrence of cervical intraepithelial neoplasia and invasive cervical cancer [8, 9]. Due to the significant regional differences in HPV infection prevalence and genotype distribution [10], understanding the local epidemiological characteristics is the basis for formulating targeted cervical cancer prevention and control strategies, including screening program optimization and HPV vaccination guidance.

Previous studies have shown that the dominant HPV genotypes in China are HPV16, 52, 58, 18 and 33 [11, 12], but there are obvious differences among different regions. Xi’an, as the core city of Northwest China, has few large-sample and long-term epidemiological data on HPV infection in recent years. This study conducted a retrospective analysis of HPV testing data collected from women attending a tertiary hospital in Xi’an over a period of approximately five years, with the aim of clarifying the prevalence, genotype distribution and age/source-specific characteristics of local HPV infection, so as to provide a scientific basis for local cervical cancer prevention and treatment. We prioritized stratification by patient source, age and sampling year for clinical and public health relevance, while age-genotype distribution patterns are further elaborated in the Discussion for comprehensive interpretation.

## Materials and methods

### Study subjects

Women aged ≥ 20 years who attended the Second Affiliated Hospital of Xi’an Jiaotong University and received HPV testing during the study period were enrolled in this retrospective analysis. Repeated testing samples from the same individual were excluded to avoid duplicate data interference. Participants were categorized by clinical source into the health examination group (HEG), inpatient group (IPG), and outpatient group (OPG). All subjects were further stratified into six predefined age brackets: 20–30, 31–40, 41–50, 51–60, 61–70, and > 70 years. Inclusion criteria: (1) Aged ≥ 20 years, with a history of sexual activity; (2) Complete clinical records; (3) Valid cervical exfoliated cell specimens collected according to standard sampling specifications. Exclusion criteria: (1) Repeated HPV testing samples from the same individual during the study period; (2) Incomplete clinical data or unqualified samples.

### Specimen collection

Cervical exfoliated cell specimens were collected by professional gynecologists following unified pre-analytical requirements. Participants were instructed to avoid vaginal intercourse, vaginal douching and topical vaginal medication for at least 24 h before sampling. Specimens were not collected during menstrual bleeding or active acute genital tract infection. Cervical exfoliated cells were obtained using a cytobrush, preserved in liquid-based cytology preservation solution, and transported to the laboratory for unified HPV nucleic acid detection within 24 h after collection.

### HPV DNA genotyping test

HPV DNA genotyping was detected by PCR-reverse dot blot hybridization using the 4800 Fully Automated Nucleic Acid Hybridization System (HEAS BioTech, Guangzhou, China) and the matching Human Papillomavirus Nucleic Acid Testing and Genotyping Reagent Kit (PCR-Reverse Dot Blot, HEAS BioTech, Guangzhou, China). DNA extraction was performed using the magnetic bead method (HEAS BioTech, Guangzhou, China) in strict accordance with the kit instructions. The test covered 28 HPV genotypes, including 18 h-HPV types (16, 18, 26, 31, 33, 35, 39, 45, 51, 52, 53, 56, 58, 59, 66, 68, 73, 82) and 10 LR-HPV types (6, 11, 40, 42, 43, 44, 54, 61, 81, 83). The appearance of blue spots at single or multiple genotypic sites was interpreted as single-type or multiple-type infection, respectively.

### Statistical analysis

Data analysis was performed using SPSS 23.0 (IBM Corp., Armonk, NY, USA), and graphs were generated using GraphPad Prism 9.0 (GraphPad Software, San Diego, CA, USA). Categorical data were expressed as frequency and rate (%). Intergroup comparison of rates was conducted using the Pearson chi-square test; multiple group comparison was performed using the chi-square partition method, with the significance level α corrected by the Bonferroni method. A two-sided *P* < 0.05 was considered statistically significant. Baseline characteristics of the study population (age distribution, patient source, year of sampling) were summarized in a dedicated table for clear presentation.

## Results

### Baseline characteristics of the study population

We characterized HPV infection patterns across three dimensions: patient source, age group, and sampling year. Patient source was designated a primary stratification variable, as it distinguishes asymptomatic screening participants from symptomatic patients at higher risk of cervical lesions. Age and annual temporal trends were analyzed to capture physiological and healthcare-related infection disparities.

A total of 33,316 women undergoing HPV testing aged ≥ 20 years were ultimately included in the present analysis. In terms of clinical source, the health examination group accounted for 31,457 participants, the outpatient group for 1,648 participants, and the inpatient group for 211 participants. Detailed age distribution, source grouping, and annual sampling volumes of the study population are summarized in Table 1 as follows: 20–30 years (*n* = 3,234, 9.71%), 31–40 years (*n* = 13,608, 40.85%), 41–50 years (*n* = 9,585, 28.77%), 51–60 years (*n* = 5,409, 16.24%), 61–70 years (*n* = 1,383, 4.15%), and > 70 years (*n* = 97, 0.29%). In terms of patient source, HEG accounted for 94.42% (31,457/33,316), OPG for 4.95% (1,648/33,316), and IPG for 0.63% (211/33,316). The number of samples collected each year was: 2019 (*n* = 2,110), 2020 (*n* = 3,465), 2021 (*n* = 5,169), 2022 (*n* = 5,693), 2023 (*n* = 7,591), 2024 (*n* = 9,288).

**Table 1:** Baseline characteristics of the study population (*N* = 33,316)

Characteristic	Category	*n*	%
Age group (years)	20–30	3,234	9.71
	31s–40	13,608	40.85
	41–50	9,585	28.77
	51–60	5,409	16.24
	61–70	1,383	4.15
	> 70	97	0.29
Patient source	Health examination group	31,457	94.42
	Outpatient group	1,648	4.95
	Inpatient group	211	0.63
Year of enrollment	2019	2,110	6.33
	2020	3,465	10.40
	2021	5,169	15.52
	2022	5,693	17.09
	2023	7,591	22.78
	2024	9,288	27.88
Total	-	33,316	100.00

### Overall HPV infection status

The overall HPV positivity rate of the 33,316 samples was 13.96% (4,650/33,316). Among the positive cases, single-type infections were the main type, accounting for 74.67% (3,472/4,650), and multiple-type infections accounted for 25.33% (1,178/4,650). Among multiple-type infections, dual infection was the most common, accounting for 71.05% (837/1,178) of multiple infections, followed by triple infection (19.19%, 226/1,178), quadruple infection (5.94%, 70/1,178), and quintuple or higher infection (3.82%, 45/1,178) (Table 2).

**Table 2:** Prevalence of single and multiple HPV infections across different age groups

Age group	Single type infection *n*, % (95% CI)	Double type infection *n*, % (95% CI)	Triple type infection *n*, % (95% CI)	Quadruple type infection *n*, % (95% CI)	Quintuple type infection and more *n*, % (95% CI)
20–30	344, 69.49% (65.20%-73.50%)	91, 18.38% (15.04%-22.17%)	40, 8.08% (5.85%-10.81%)	9, 1.82% (0.84%-3.43%)	11, 2.22% (1.11%-3.94%)
31–40	1,245, 77.52% (75.36%-79.54%)	282, 17.56% (15.70%-19.57%)	56, 3.49% (2.65%-4.50%)	14, 0.87% (0.48%-1.46%)	9, 0.56% (0.26%-1.07%)
41–50	976, 78.90% (76.62%-81.00%)	193, 15.60% (13.65%-17.74%)	53, 4.28% (3.22%-5.56%)	11, 0.89% (0.45%-1.59%)	4, 0.32% (0.09%-0.82%)
51–60	701, 70.59% (67.61%-73.40%)	200, 20.14% (17.72%-22.77%)	58, 5.84% (4.47%-7.50%)	21, 2.12% (1.31%-3.23%)	13, 1.31% (0.70%-2.22%)
61–70	196, 64.90% (59.20%-70.20%)	66, 21.85% (17.30%-27.00%)	18, 5.96% (3.56%-9.24%)	15, 4.97% (2.81%-8.05%)	7, 2.32% (0.94%-4.72%)
> 70	10, 58.82% (32.90%-81.56%)	5, 29.41% (11.30%-55.36%)	1, 5.88% (0.15%-28.68%)	0, 0.00% (0.00%-19.51%)	1, 5.88% (0.15%-28.68%)
Total	3,472, 74.67% (73.39%-75.91%)	837, 18.00% (16.88%-19.17%)	226, 4.86% (4.26%-5.52%)	70, 1.51% (1.18%-1.90%)	45, 0.97% (0.71%-1.29%)

Note: Data are presented as n, % (95% Clopper–Pearson exact confidence interval). Percentages are calculated as the number of individuals with a specific infection type divided by the total number of infected individuals in the corresponding age group, multiplied by 100. Pearson χ^2^ test was used to compare the distribution of single and multiple HPV infections across different age groups (χ^2^ = 105.65, df = 20, *P* < 0.001). HPV, human papillomavirus

### Temporal trends in HPV infection positivity by patient source

There were significant differences in HPV infection rates among different patient sources (*P* < 0.001). The infection rate of HEG was 12.67% (3,985/31,457), that of IPG was 20.85% (44/211), and that of OPG was the highest (37.68%, 621/1,648). The annual HPV infection rate of each group showed a fluctuating upward trend (Table 3, *P* < 0.001 for interannual comparison). HEG’s infection rate was 11.52% in 2019, decreased to the lowest (7.25%) in 2021, and then rose to 16.62% in 2023 and 16.20% in 2024. IPG had no positive cases before 2022, and the infection rates were 22.22% (2022), 21.43% (2023) and 21.79% (2024) after 2022. OPG’s infection rate was low before 2021, increased significantly to 18.75% in 2021, and continued to rise to 33.33% (2022), 50.00% (2023) and 37.82% (2024). The overall HPV infection rate in 2024 (19.94%) was significantly higher than that in 2019 (11.52%) (*P* < 0.001).

**Table 3:** Prevalence of HPV infection among Health examination group (HEG), Inpatient group (IPG), and Outpatient group (OPG) from 2019 to 2024

Source	2019 *n*, % (95% CI)	2020 *n*, % (95% CI)	2021 *n*, % (95% CI)	2022 *n*, % (95% CI)	2023 *n*, % (95% CI)	2024 *n*, % (95% CI)
HEG	243, 11.52% (10.18%-12.98%)	329, 9.50% (8.54%-10.53%)	373, 7.25% (6.55%-8.00%)	562, 9.96% (9.17%-10.80%)	1,255, 16.62% (15.80%-17.46%)	1,223, 16.20% (15.39%-17.03%)
IPG	0, 0.00% (0.00%-3.69%)	0, 0.00% (0.00%-3.69%)	0, 0.00% (0.00%-3.69%)	2, 22.22% (2.81%-60.00%)	3, 21.43% (4.66%-47.57%)	39, 21.79% (16.00%-28.57%)
OPG	0, 0.00% (0.00%-3.69%)	0, 0.00% (0.00%-3.69%)	3, 18.75% (4.04%-43.45%)	14, 33.33% (19.58%-49.57%)	14, 50.00% (30.66%-69.34%)	590, 37.82% (35.42%-40.26%)
Total	243, 11.52% (10.18%-12.98%)	329, 9.50% (8.54%-10.53%)	376, 7.27% (6.58%-8.02%)	578, 10.15% (9.37%-11.00%)	1,272, 16.76% (15.94%-17.61%)	1,852, 19.94% (19.04%-20.86%)

Note: Data are presented as n, % (95% CI). 95% confidence intervals were calculated via the Clopper–Pearson exact binomial method. Pearson χ^2^ test was used to compare the overall HPV infection rates among the three groups (χ^2^ = 296.30, df = 2, *P* < 0.001). HPV, human papillomavirus.s

### HPV genotype distribution

**Fig. 1:**
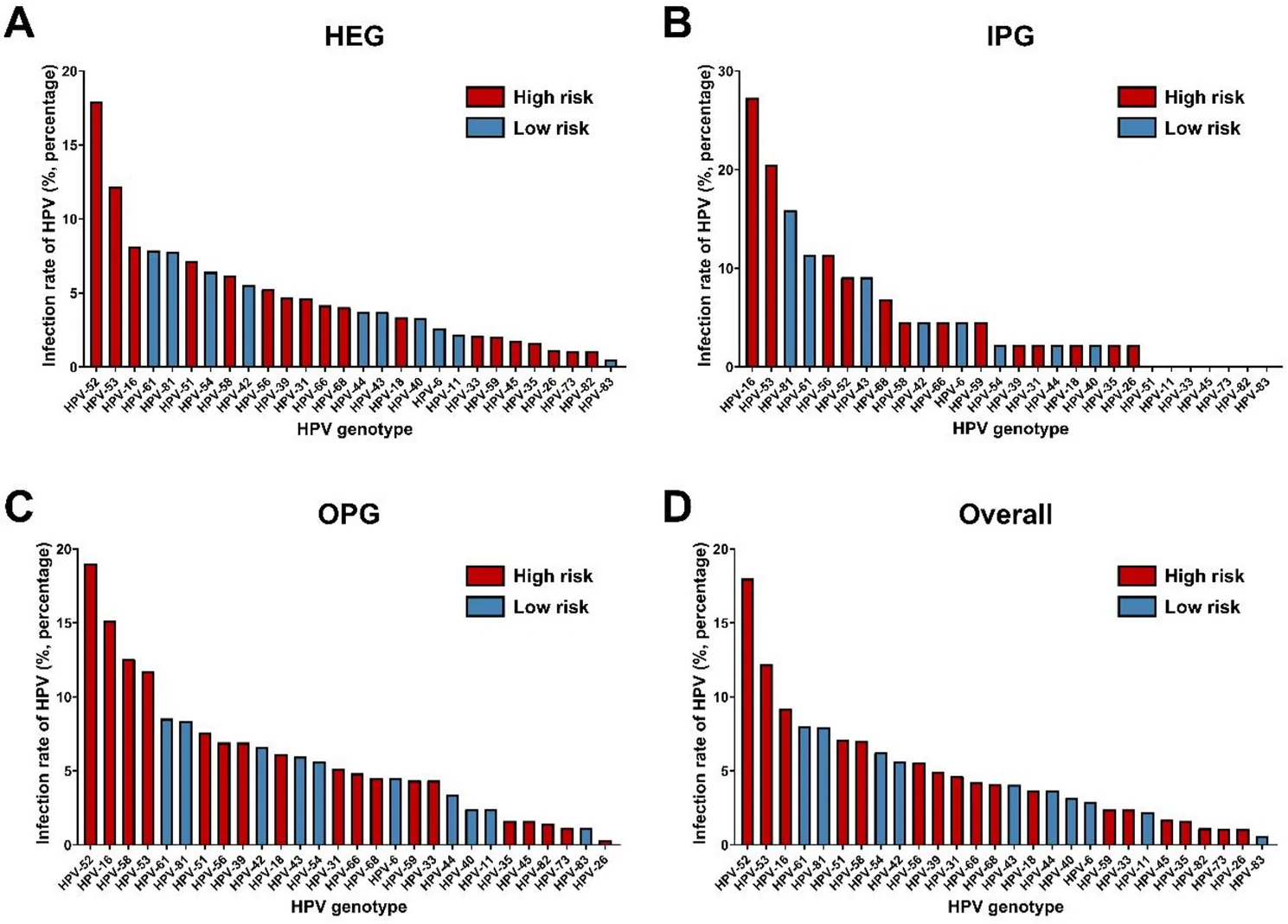
Distribution of HPV genotypes among women undergoing HPV testing from different sources. **A**: Health Examination Group (HEG); **B**: Inpatient Group (IPG); **C**: Outpatient Group (OPG); **D**: Overall study population

Among all HPV-positive patients, the top six genotypes with the highest infection rates were HPV52 (18.00%, 837/4,650), HPV53 (12.19%, 567/4,650), HPV16 (9.23%, 429/4,650), HPV61 (8.00%, 372/4,650), HPV81 (7.94%, 369/4,650), and HPV51 (7.12%, 331/4,650) (Fig. 1). The dominant genotypes varied by patient source: HEG: The top five genotypes were HPV52 (17.94% of positive cases), HPV53 (12.17%), HPV16 (8.11%), HPV61 (7.88%), and HPV81 (7.78%); IPG: The top five genotypes were HPV16 (27.27% of positive cases), HPV53 (20.45%), HPV81 (15.91%), HPV61 (11.36%), and HPV56 (11.36%); OPG: The top five genotypes were HPV52 (19.00% of positive cases), HPV16 (15.14%), HPV58 (12.56%), HPV53 (11.76%), and HPV61 (8.53%). HR-HPV was the main type of infection in all three groups, accounting for more than 80% of positive cases. Specifically, the detection rate of HPV16 in IPG and OPG was significantly higher than that in HEG (*P* < 0.05).

### HPV infection prevalence by age group

**Fig. 2:**
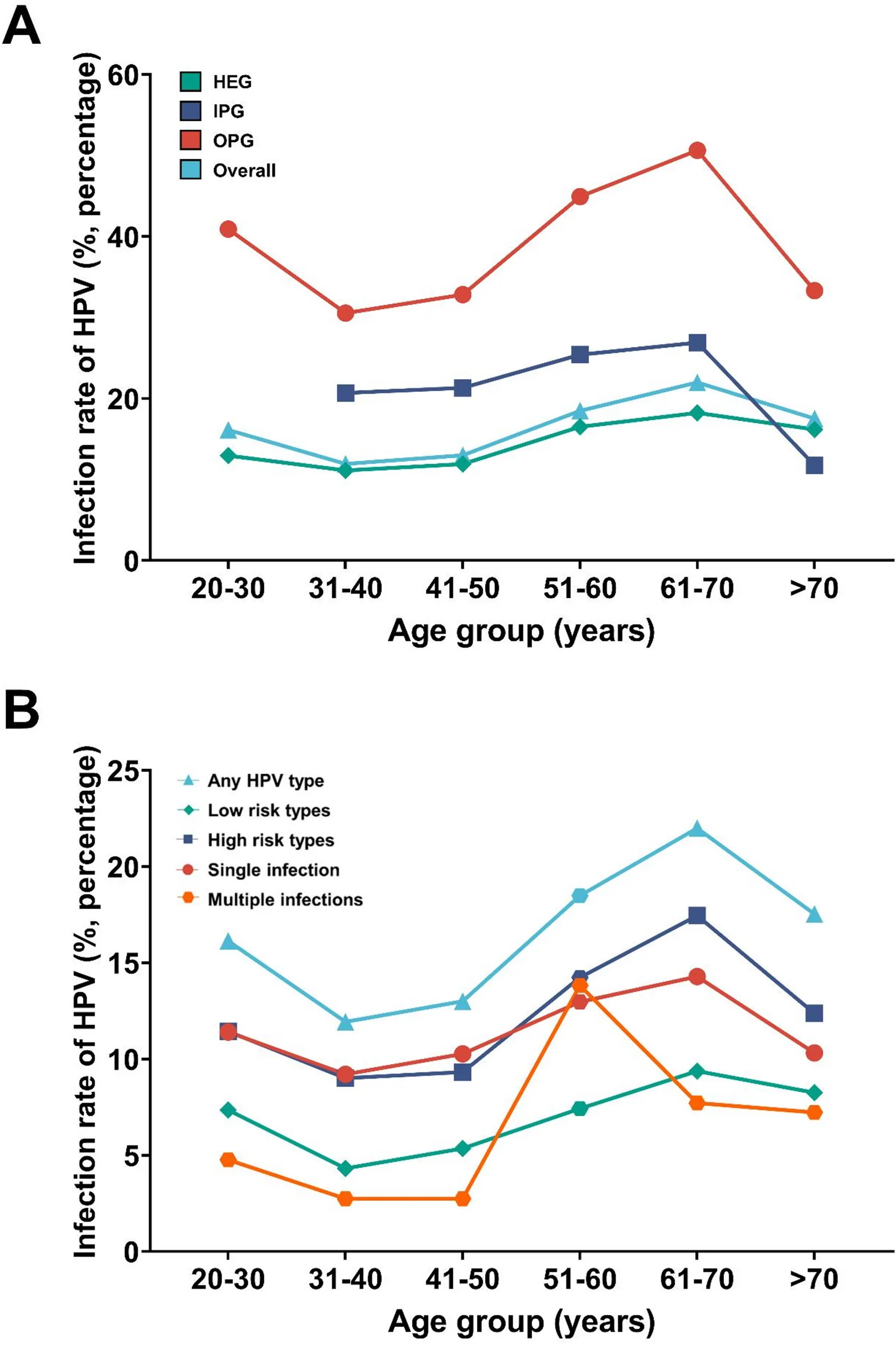
Prevalence of HPV infection by age group. **A**: HPV positivity rates in HEG, IPG, OPG, and overall population; **B**: Proportions of any HPV infection, low‑risk HPV infection, high‑risk HPV infection, single infection, and multiple infection across age groups. HEG, Health Examination Group; IPG, Inpatient Group; OPG, Outpatient Group

There were significant differences in the overall HPV infection rate among different age groups (*P* < 0.001) (Fig. 2A). Stratified by patient source within each age bracket, HPV positivity was consistently highest in the outpatient group across nearly all age strata, while the overall population-wide HPV prevalence peaked in women aged 61–70 years (21.99%), followed by the 51–60 years group (18.48%), and reached the lowest level in the 31–40 years group (11.93%). Distinct age-specific patterns were observed for the internal composition of infection subtypes among HPV-positive individuals (Fig. 2B; Table 2). The proportion of single-type infections among HPV-positive women was highest in the 41–50 years group (78.90%). Dual infections occupied the largest share of positive cases in women aged 61–70 years (21.85%). Furthermore, participants aged 61–70 years exhibited the greatest proportion of triple or higher-order multiple infections at 14.28%, which was statistically higher compared with all other age strata (*P* < 0.05).

### Temporal trends of HPV infection types

**Fig. 3:**
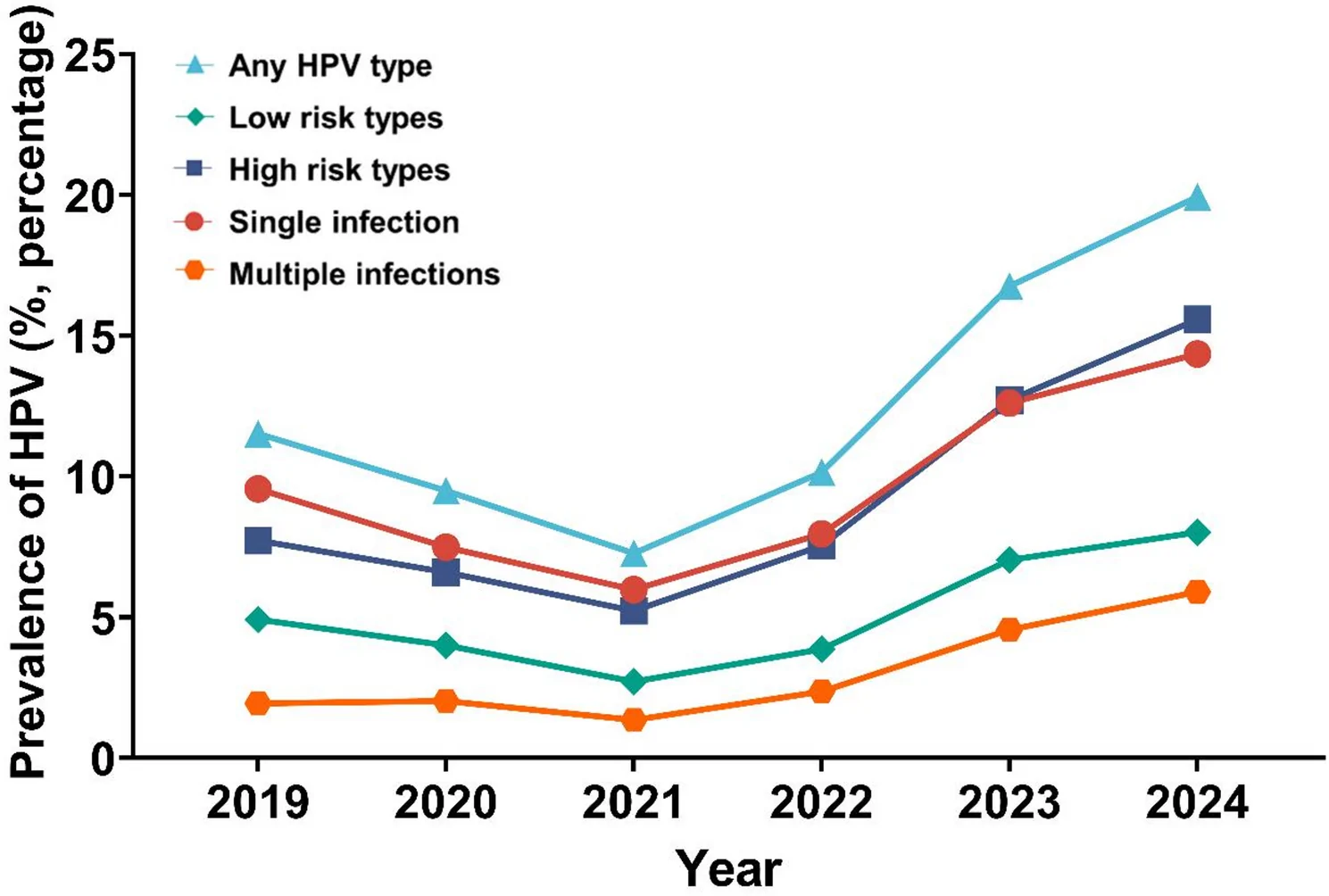
Temporal trends in the prevalence of different HPV infection types from 2019 to 2024. The overall HPV positivity rate (any HPV type) and high-risk type HPV prevalence showed a fluctuating upward trend, reaching 19.94% and 15.58% in 2024, respectively. Single-type infection remained the dominant pattern, while multiple infections and low-risk type HPV prevalence increased gradually but remained at lower levels throughout the study period

Consistent with the annual infection trends stratified by patient source shown above, the aggregate HPV positivity rose markedly from 11.52% in 2019 to 19.94% in 2024 (Table 3). The change trend of HR-HPV infection rate was consistent with the overall trend, rising from 7.72% in 2019 to 15.58% in 2024 (an increase of 101.81%). Single-type HPV infection remained the primary infection pattern, with its prevalence increasing from 9.56% in 2019 to 14.35% in 2024 (a 50.10% increase) (Fig. 3). The prevalence of LR-HPV remained consistently low (4.00%-8.01%) during the study period, with no obvious interannual fluctuation trend.

## Discussion

This study analyzed the epidemiological characteristics of HPV infection in 33,316 women in Xi’an from 2019 to 2024, and found that the overall HPV positivity rate was 13.96%, with single-type infection as the main type, and HPV52, HPV53 and HPV16 as the dominant genotypes. The infection rate varied significantly by patient source and age, and showed a fluctuating upward trend in the past 6 years. These results are consistent with the characteristics of regional HPV infection in Northwest China, and also reflect the unique epidemiological changes of HPV infection in Xi’an after the COVID-19 pandemic.

### Genotype distribution characteristics and regional comparison

HPV52, HPV53 and HPV16 were the predominant genotypes identified in our cohort. This pattern partially aligns with national multicenter epidemiological data reporting HPV16, 52, 58 and 18 as top prevalent strains [10–12], yet significant regional divergence was observed: HPV53 was far more frequently detected in Xi’an, while HPV18 and HPV58 showed relatively low prevalence. Such geographic discrepancies may stem from regional genetic backgrounds, population mobility and local sexual behavioral patterns in Northwest China. In addition, HPV16 positivity was markedly higher among inpatients and outpatients than asymptomatic screening participants, consistent with prior evidence that HPV16 confers the highest risk of cervical lesions and malignant disease [13, 14]. Specifically, HPV53 was abundant across all patient subgroups, marking a unique epidemiological signature of this local cohort. Multiple regional Shaanxi studies have documented widespread HPV53 circulation [15–17]. However, our cross-sectional screening data cannot verify its long-term viral persistence or independent oncogenic capacity. Longitudinal cohort research tracking HPV53-related lesion progression is required to quantify its clinical risk. Furthermore, current nine-valent vaccines do not cover HPV53, highlighting the need for further population-level research on vaccine protection against locally prevalent genotypes. Regional genetic susceptibility, distinct sexual behavior patterns and localized viral transmission chains have been hypothesized to drive the dominance of HPV53 in Northwest China based on genomic epidemiological literature. These population-level mechanisms remain speculative and cannot be validated by our single-center cross-sectional dataset. Objective genotype distribution results derived from the present study should be clearly distinguished from external literature-based conjectures to prevent overinterpretation of observational epidemiological data.

### Age and patient source characteristics of HPV infection

Specifically, patient source was prioritized as a core, primary stratification variable rather than a secondary exploratory factor in this hospital-based retrospective study, and we set greater emphasis on this factor for robust clinical rationale. Grouping participants by clinical presentation (asymptomatic screening attendees vs. symptomatic outpatient/inpatient women) directly distinguishes individuals with pre-existing gynecological complaints or suspected cervical lesions from low-risk asymptomatic populations. Significant gaps in overall HPV positivity and HPV16 detection rates between the three source subgroups further confirm that patient source was significantly associated with elevated risk of severe HPV-related cervical lesions. This stratification may provide useful reference information for hospital-based hierarchical risk stratification and enhanced follow-up planning for symptomatic patients with high clinical suspicion, consistent with our primary study objective of generating locally applicable cervical cancer prevention evidence. Although age-genotype distribution offers population-based epidemiological context, it may not directly serve as standalone evidence to guide real-time frontline clinical triage decisions. To offset the relatively concise genotype-age stratified results, we further explored age-specific variations in dominant HR-HPV genotypes to fully characterize regional HPV epidemiological features.

Significant age-related differences in HPV prevalence were observed. The highest infection rate was found in the 61–70 years group (21.99%), followed by the 51–60 years group (18.48%) and the > 70 years group (17.53%). The lowest rate was observed in the 31–40 years group (11.93%), with rates of 16.13% in the 20–30 years group and 13.00% in the 41–50 years group. This bimodal or elderly-peaking pattern suggests that women in the perimenopausal and postmenopausal stages carry the highest HPV burden in this study population. Elderly women generally experience declined mucosal immunity, reduced ability to clear persistent HPV infection, and potential viral reactivation due to hormonal and immunological changes after menopause, which may contribute to the elevated infection rate in the 61–70 years group [18, 19]. Although the 20–30 years group did not show the highest overall prevalence, young women still represent a high-risk cohort for incident infection due to early sexual activity and relatively immature local immune function [12]. Therefore, targeted interventions including HPV vaccination and regular screening remain critical for young women.

The HPV positivity rate was significantly higher among outpatients and inpatients than in the health examination population, which can be explained by referral bias. Outpatients and inpatients mostly presented with gynecological symptoms or suspected cervical lesions, leading to a higher likelihood of HPV infection. In contrast, the health examination population consisted of asymptomatic individuals with a lower baseline risk. The notably higher detection rate of HPV16 in inpatients further supports that HPV16 is strongly associated with severe cervical lesions, which is of great clinical importance for guiding risk-based screening and intensive follow-up of high-risk populations.

### Temporal trend of HPV infection and the impact of COVID-19 pandemic

Our 6-year observational data demonstrated fluctuating HPV positivity rates from 2019 to 2024, with a lower detection level in 2021 and a gradual increase in overall positivity thereafter. Several plausible hypotheses based on previous epidemiological studies may help explain this temporal pattern, including pandemic-induced restrictions on routine gynecological screening, delayed medical seeking behavior, accumulated screening demands after healthcare service recovery, and potential population behavioral or immune alterations during and after the pandemic [20, 21]. These are plausible hypotheses proposed based on previous published studies, which cannot be directly validated by the present retrospective cross-sectional data.

Descriptive disparities in temporal trends were observed between HR-HPV and LR-HPV genotypes in our cohort. However, these divergent annual prevalence changes only represent observational differences and do not confirm a stronger pandemic-specific effect on HR-HPV circulation. Further longitudinal population-based studies are required to verify genotype-specific temporal variation and underlying driving mechanisms.

### Limitations and future research directions

This study has several limitations: (1) This single-center hospital-based retrospective cohort carries inherent selection bias. All participants were recruited from one tertiary hospital in Xi’an; therefore, the findings cannot fully represent the general female population across the whole city, restricting external validity; (2) Sample imbalance exists across patient source subgroups: around 94.42% of women undergoing HPV testing belonged to the health examination group, with only small proportions of outpatient and inpatient participants. Cross-classification by age, patient source and sampling year generated many strata with minimal or zero HPV-positive cases, which would trigger quasi-complete separation and produce biased, uninterpretable outputs if multivariable logistic regression was performed. Consequently, all intergroup comparisons are unadjusted crude positivity rates, and confounding interactions among the three core variables cannot be statistically eliminated. This uneven enrollment also overrepresents asymptomatic screening women and weakens the generalizability of results to symptomatic gynecological cohorts; (3) Critical behavioral and clinical confounding variables were not recorded in routine hospital testing records, including sexual activity history, smoking status, and underlying immunosuppressive conditions. These unmeasured factors may independently modify HPV infection risk and cannot be controlled in our univariate analyses. In addition, complete HPV vaccination records were unavailable for all participants, preventing us from evaluating how vaccination shapes local HPV prevalence and genotype distribution patterns; (4) Only HPV testing results were extracted for analysis, without matched cervical histopathological data. We cannot quantify the independent correlation between distinct HPV genotypes (including locally prevalent HPV53) and the severity of cervical lesions or malignant progression risk. For future studies, multi-center cohorts with balanced enrollment of screening, outpatient and inpatient women are required to validate our epidemiological observations. Subsequent studies will systematically collect missing covariates such as sexual behavior, smoking, immunosuppression and HPV vaccination history, and incorporate histopathological lesion outcomes to enable multivariate confounding adjustment. Further long-term follow-up work is also planned to explore the oncogenic potential of regional dominant genotypes like HPV53, as well as the regulatory effects of immune markers (e.g., mannose-binding lectin, MBL) on persistent HPV infection and cervical carcinogenesis, to build more robust evidence for local cervical cancer prevention strategies.

### Public health implications

Based on stratified HPV infection profiles observed in this single-center cohort, we put forward local cervical cancer prevention references for Xi’an, with all recommendations requiring further prospective validation: (1) Prioritize routine HPV vaccination for females aged 9–14 years before sexual debut and expand uptake among women aged 20–25 years, to reduce transmission of dominant genotypes HPV52 and HPV16; (2) Stratified screening schemes may be considered for local tertiary hospitals, focusing surveillance on high-risk groups including older postmenopausal women and symptomatic gynecological attendees. We refrain from definitive suggestions on elevated screening frequency, as optimal screening intervals cannot be determined without long-term lesion outcome data. Conditional intensified follow-up could be implemented for patients with multiple HPV infections under clinical judgment; this proposal remains preliminary because our dataset lacks matched histopathology results to confirm independent links between multi-genotype infection and severe cervical lesions; (3) Establish regular local HPV epidemiological surveillance to track dynamic shifts in positivity and genotype composition, providing foundational data for future adjustments to regional screening and immunization policies; (4) Sustain population health education to improve public awareness of HPV screening and vaccination across all age brackets.

## Data Availability

All data generated or analyzed during this study are included in this published article (and its supplementary materials).
